# Assimilative and non-assimilative color spreading in the watercolor configuration

**DOI:** 10.3389/fnhum.2014.00722

**Published:** 2014-09-19

**Authors:** Eiji Kimura, Mikako Kuroki

**Affiliations:** ^1^Department of Psychology, Faculty of Letters, Chiba UniversityChiba-shi, Chiba, Japan; ^2^Graduate School of Humanities and Social Sciences, Chiba UniversityChiba-shi, Chiba, Japan

**Keywords:** watercolor effect, assimilation, color spreading, luminance contrast, visual illusion

## Abstract

A colored line flanking a darker contour will appear to spread its color onto an area enclosed by the line (watercolor effect). The watercolor effect has been characterized as an assimilative effect, but non-assimilative color spreading has also been demonstrated in the same spatial configuration; e.g., when a black inner contour (IC) is paired with a blue outer contour (OC), yellow color spreading can be observed. To elucidate visual mechanisms underlying these different color spreading effects, this study investigated the effects of luminance ratio between the double contours on the induced color by systematically manipulating the IC and the OC luminance (Experiment 1) as well as the background luminance (Experiment 2). The results showed that the luminance conditions suitable for assimilative and non-assimilative color spreading were nearly opposite. When the Weber contrast of the IC to the background luminance (IC contrast) was smaller in size than that of the OC (OC contrast), the induced color became similar to the IC color (assimilative spreading). In contrast, when the OC contrast was smaller than or equal to the IC contrast, the induced color became yellow (non-assimilative spreading). Extending these findings, Experiment 3 showed that bilateral color spreading, i.e., assimilative spreading on one side and non-assimilative spreading on the other side, can also be observed in the watercolor configuration. These results suggest that the assimilative and the non-assimilative spreading were mediated by different visual mechanisms. The properties of the assimilative spreading are consistent with the model proposed to account for neon color spreading (Grossberg and Mingolla, 1985) and extended for the watercolor effect (Pinna and Grossberg, 2005). However, the present results suggest that additional mechanisms are needed to account for the non-assimilative color spreading.

## Introduction

**Figure 1:**
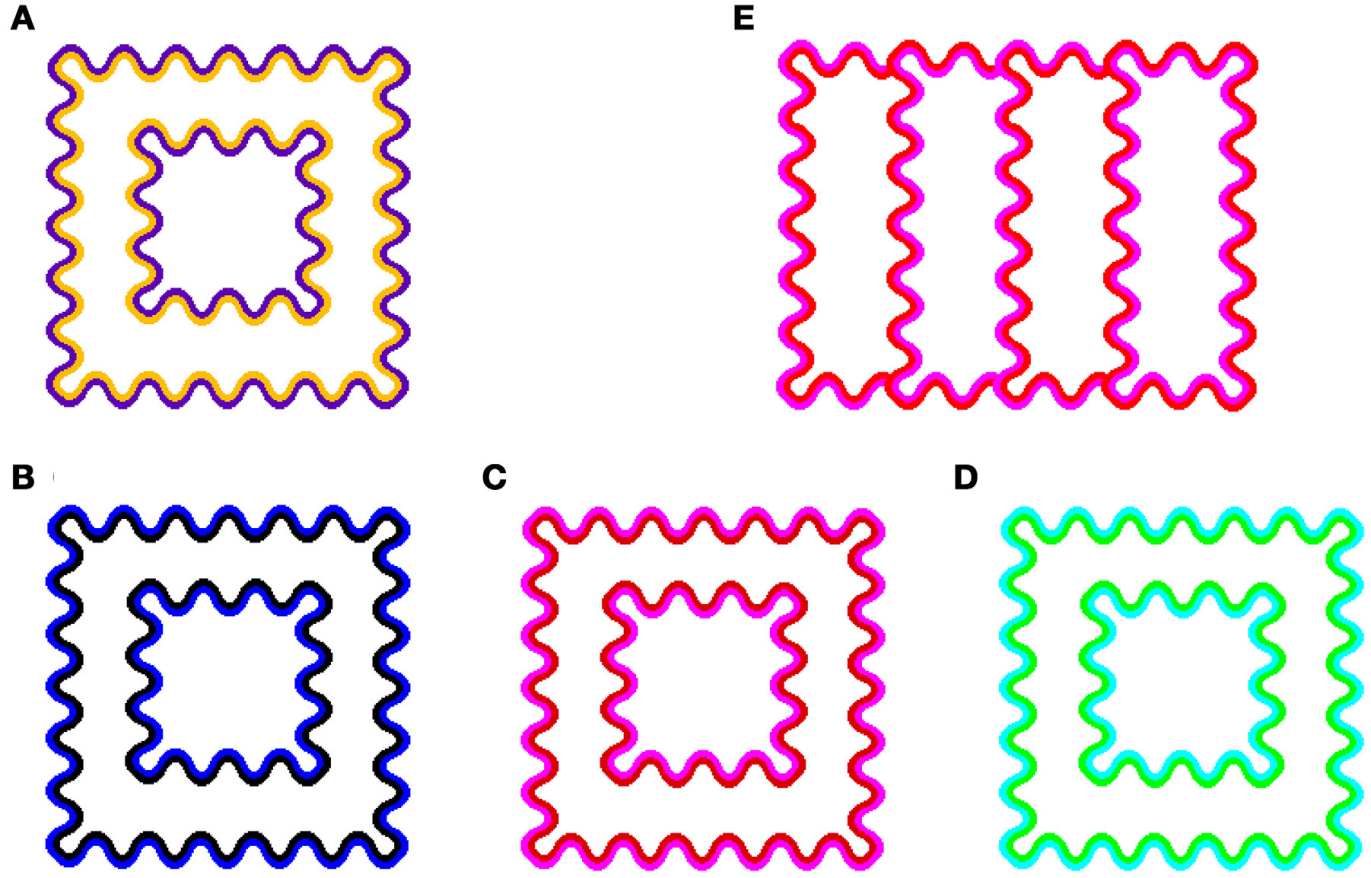
**Demonstrations of color spreading in the watercolor configuration. (A)** Conventional assimilative color spreading in an orange-purple color combination (Pinna et al., 2001). **(B–D)** Examples of non-assimilative yellow color spreading. A black-blue color combination **(B)** was first demonstrated in Pinna (2006). Red-magenta **(C)** and green-cyan **(D)** as well as the black-blue combinations were demonstrated as “the paradoxical watercolor effect” in Kitaoka (2007). **(E)** A four-column configuration used in Experiment 3.

A colored line running parallel and contiguous to a darker contour will appear to spread its color onto a white area enclosed by the line (Figure 1A). This phenomenon is called the watercolor effect (Pinna et al., 2001). The watercolor effect involves long-range color spreading over distances of up to 45° (Pinna et al., 2001). Many color combinations can produce the effect (Pinna et al., 2001), although a combination of complementary colors seems to produce stronger chromatic induction (Pinna et al., 2001; Devinck et al., 2006b). Moreover, it has been shown that the luminance ratio of the double contours plays a critical role in producing color spreading (Pinna et al., 2001). Nevertheless, the color spreading does not involve large brightness changes (Devinck et al., 2005). Recent studies revealed that the achromatic watercolor effect does exist and is measurable, but the magnitude is small (Takashima, 2008; Cao et al., 2011). Regarding the effects of luminance conditions, previous studies showed that the spreading was very weak when the luminance of the outer contour (OC) was in between the luminances of the inner contour (IC) and of the background (Devinck et al., 2005; Cao et al., 2011). The magnitude of color spreading increased with the luminance ratio of the IC to the OC (Devinck et al., 2005; Devinck and Knoblauch, 2012). Based on these findings, Devinck et al. (2005) suggested that the watercolor effect is mediated by luminance-dependent color mechanisms.

In the watercolor effect, the induced color is generally similar to the IC color, and thus the effect has been characterized as a color assimilation effect (e.g., Pinna et al., 2001; Devinck et al., 2005, 2006b). For example, in a typical watercolor display (orange-purple combination) shown in Figure 1A, the corridor region looks uniformly tinted by the orange color of the IC. However, a non-assimilative type of color spreading has also been reported. Pinna (2006) reported that the induced color can be complementary to the *OC* color, when the IC was achromatic and the OC was chromatic (see Figure 1B). In addition, Kitaoka (2007) demonstrated that a red-magenta (Figure 1C) and a green-cyan combination (Figure 1D) also produce similar non-assimilative (yellow) color spreading. These findings suggest that visual mechanisms other than the ones mediating color assimilation also contribute to the watercolor effect.

Similar assimilative and non-assimilative effects have also been demonstrated in neon color spreading. For example, Van Tuijl (1975) showed that when sections of a lattice composed of horizontal and vertical black lines are replaced by colored segments, the color appears to spread among the segments, producing the impression of a transparent veil overlapping the lattice (neon color spreading). Moreover, in the opposite color combination, i.e., when the sections of a colored lattice are replaced by black segments, the complementary color of the lattice now appears to spread among the segments. These observations have been extended to other color combinations such as red-magenta and green-cyan (Bressan, 1995; da Pos and Bressan, 2003; Sohmiya, 2004). In fact, these studies were referred by Kitaoka (2007).

Bressan (1995) proposed that the induced color in the neon color configuration can be accounted for by a mixture between the color of the segment and the complementary color of the lattice. This explanation is based on a computational model developed by Grossberg and Mingolla (1985), which distinguishes two parallel processing systems, the boundary contour system (BCS) and the feature contour system (FCS). The BCS generates invisible boundaries, while the FCS fills the space limited by these boundaries with color and brightness. In the BCS, signal coming from the lattice lines in the neon color configuration amplifies signals for perpendicular boundaries at the junctions between lattice lines and segments, creating illusory contours which delimit the segment region. At the same time, the signal from the lattice lines inhibits signals for the contiguous aligned segments, making them permeable. In the FCS, the color of the segments flows out through the permeable boundaries, and spread until it reaches the illusory contours. In addition, Bressan (1995) proposed as an elaboration of Grossberg and Mingolla's (1985) model that, in the FCS, as a manifestation of simultaneous color contrast, the lattice lines induce complementary color into the segments, which also flows out through the weakened boundaries of the segments. As a result, the induced color is an additive mixture between the color diffused from the inhibited segments and the complementary color of the lattice. da Pos and Bressan (2003) empirically confirmed that the neon color effect can be predicted by a combination of color diffusion and complementary color induction. Bressan's proposal is also consistent with the findings that neon color spreading is strongest if the colors of the inducers and segments are complementary to each other, and weaker if they are similar (Ejima et al., 1984).

To account for non-assimilative color spreading in the watercolor configuration, the contribution of the OC color, as well as of the IC color, to the chromatic induction is crucial. Pinna and Grossberg (2005) has already extended the model by Grossberg and Mingolla (1985) to account for color spreading from lines flanking each other in the watercolor effect, and discussed similarities and dissimilarities between the watercolor and the neon color effects. Thus, Bressan's explanation may be applicable to the watercolor effect as well. However, the effects of luminance ratio between the double contours in the watercolor configuration need to be taken into account. Grossberg and Mingolla's (1985) model assumes that the strength of boundaries is sensitive to contrast but insensitive to contrast polarity. In other words, the strength of boundaries can be associated with the size (absolute value) of the Weber contrast of the contour to the background luminances. The Weber contrast is defined as (L_C_-L_Bkg_)/L_Bkg_, where L_C_ is the contour luminance and L_Bkg_ is the background luminance, and thus when L_C_ is smaller or larger than L_Bkg_, the size of the Weber contrast becomes larger. According to Grossberg and Mingolla's (1985) model, higher-contrast boundaries inhibit spatially contiguous lower-contrast boundaries more than conversely, thereby enabling color to flow out through weaker boundaries. In the typical orange-purple watercolor display on a white background, the darker purple OC has a larger luminance contrast than the brighter orange IC, and thus the model predicts that the boundaries of the lower-contrast orange/white edges would be weakened than those of the higher-contrast purple/white edges, so that orange color flows out through boundaries into the region enclosed by the orange IC. The prediction is consistent with the empirical findings (Devinck et al., 2005; Cao et al., 2011; Devinck and Knoblauch, 2012; Coia et al., 2014). In particular, Cao et al. (2011) showed that, in the achromatic watercolor effect, darker spreading can be induced from the IC that had lower luminance than the background regardless of luminance contrast polarity for the OC against the background. That is, a darker perception was induced in the test region when the OC had a higher or lower luminance than both the IC and the background.

For non-assimilative color spreading, however, some refinement of the model seems necessary. Based on phenomenal observations, Kitaoka (2007) reported that non-assimilative color spreading becomes stronger when the OC has higher luminance than the IC and thus exhibits lower contrast to a white background. In this case, the spatial competition within the BCS would result in the inhibition of the boundaries of the lower-contrast OC edges, so that the OC color is expected to spread out. In contrast to the prediction, what is observed is, when the IC is red and the OC is magenta for instance, yellow color spreading on the *opposite* side of the contours; i.e., in the region delineated by the red contour (see Figure 1C). Moreover, Devinck et al. (2005) showed that, using a typical orange-purple combination in the watercolor display, the induced color systematically changed with the luminance ratio between the IC and the OC. When the luminance of the orange IC was higher and the luminance ratio of the IC to the OC was high, color spreading was assimilative (i.e., orange). When the luminance ratio was decreased, the induced color became increasingly dissimilar to the IC color. And then, when the IC luminance became much lower than the OC luminance (e.g., 1/4 to 1), the induced color became yellow and similar to the complementary color of the purple OC. These results suggest that the luminance ratio between the double contours plays a critical role in determining the induced color in the watercolor effect.

To improve the understanding of color spreading in the watercolor configuration, the present study further explored the effects of luminance conditions on the induced color in the watercolor configuration. We systematically manipulated the IC and the OC luminances (Experiment 1) as well as the background luminance (Experiment 2). We are particularly interested in the questions of what luminance conditions are suitable for assimilative and non-assimilative color spreading, and of whether the induced color can always be accounted for by an additive mixture between the IC color and the complementary color of the OC.

## Experiment 1

In Experiment 1, we examined the effects of the luminance ratio between the IC and the OC on the induced color in the watercolor configuration. In addition to a typical orange-purple combination, we also used a red-magenta combination that has been used to demonstrate non-assimilative color spreading. As the non-assimilative color spreading has been demonstrated only with a few color combinations (i.e., red-magenta, green-cyan, or blue-black), we explored a possibility that particular color combinations play an important role in producing the non-assimilative color spreading. Furthermore, in order to possibly differentiate the contributions of the IC and the OC to the induced color, the IC (or OC) luminance was manipulated when the OC (or IC) luminance was fixed.

### Methods

#### Observers

Three observers (including the second author) participated in Experiment 1. All observers had normal or corrected-to-normal visual acuity and normal color vision as assessed with Ishihara pseudo-isochromatic plates. The two observers other than the author were naïve regarding the purpose of the experiment. All observers who participated in this and following experiments gave informed consent after thorough explanation of the procedures.

#### Apparatus

The stimuli were generated by a Cambridge Research Systems VSG 2/5 graphic card and displayed on a 21-in. Sony color monitor (GDM F500R) with a pixel resolution of 1280 × 962 and a frame rate of 80 Hz. The intensity of each phosphor could be varied with 15-bit resolution. Spectroradiometric calibration was performed on three phosphors of the monitor with a Minolta CS-1000 spectroradiometer and an LS-100 luminance meter. The Psychophysics toolbox extensions for Matlab were used in the phosphor calibrations and colorimetric calculations (Brainard, 1997; Pelli, 1997). A chin and forehead rest was used to maintain a viewing distance of 86 cm. The experiment was run in a dark room.

#### Stimuli

The stimulus was composed of outer (3.7°) and inner (2.1°) squares, and color spreading in the corridor area (test area) was investigated. The squares were delineated by sinusoidally shaped double contours (1.9 c/degree, 0.4° peak-to-trough amplitude, and 4.5 min thick). The luminance of the white background was 60 cd/m^2^ and its chromaticity coordinate was *u*′ = 0.1978, *v*′ = 0.4683 in the CIE u′v′ chromaticity diagram. In the u′v′ chromaticity diagram, +u′ vs. −u′ directions relative to white roughly correspond to red vs. green directions, respectively, whereas +v′ vs. −v′ directions roughly correspond to yellow vs. blue directions, respectively.

Two combinations of the IC and OC colors were used. One was an orange IC and purple OC combination (see Figure 1A). The chromaticity coordinates were *u*′ = 0.2297, *v*′ = 0.5068 for orange and *u*′ = 0.2047, *v*′ = 0.4188 for purple. The other was a red IC and magenta OC combination (see Figure 1C). The chromaticity coordinates were *u*′ = 0.2463, *v*′ = 0.4804 for red and *u*′ = 0.2313, *v*′ = 0.4312 for magenta. Euclidean distance between the chromaticity coordinates between each of four colors and the white background was 0.05 in the u′v′ chromaticity diagram and thus their saturation were similar.

The luminance ratio of the IC to the OC was manipulated in two different fashions (Table 1). In one stimulus condition, the IC luminance was fixed to 20 cd/m^2^ and the OC luminance was varied from 5 to 55 cd/m^2^ (variable OC luminance condition). In the other condition, the luminance relation was reversed; the OC luminance was fixed to 20 cd/m^2^ and the IC luminance was varied from 5 to 55 cd/m^2^ (variable IC luminance condition). As in Devinck et al. (2005), the luminance conditions in Experiment 1 will be described in terms of the luminance ratio between the IC and the OC (IC/OC), but see Table 1 for other luminance measures. The IC/OC luminance ratio was varied from 0.25 to 4.0 (−0.6 to 0.6 in log units).

**Table 1:**
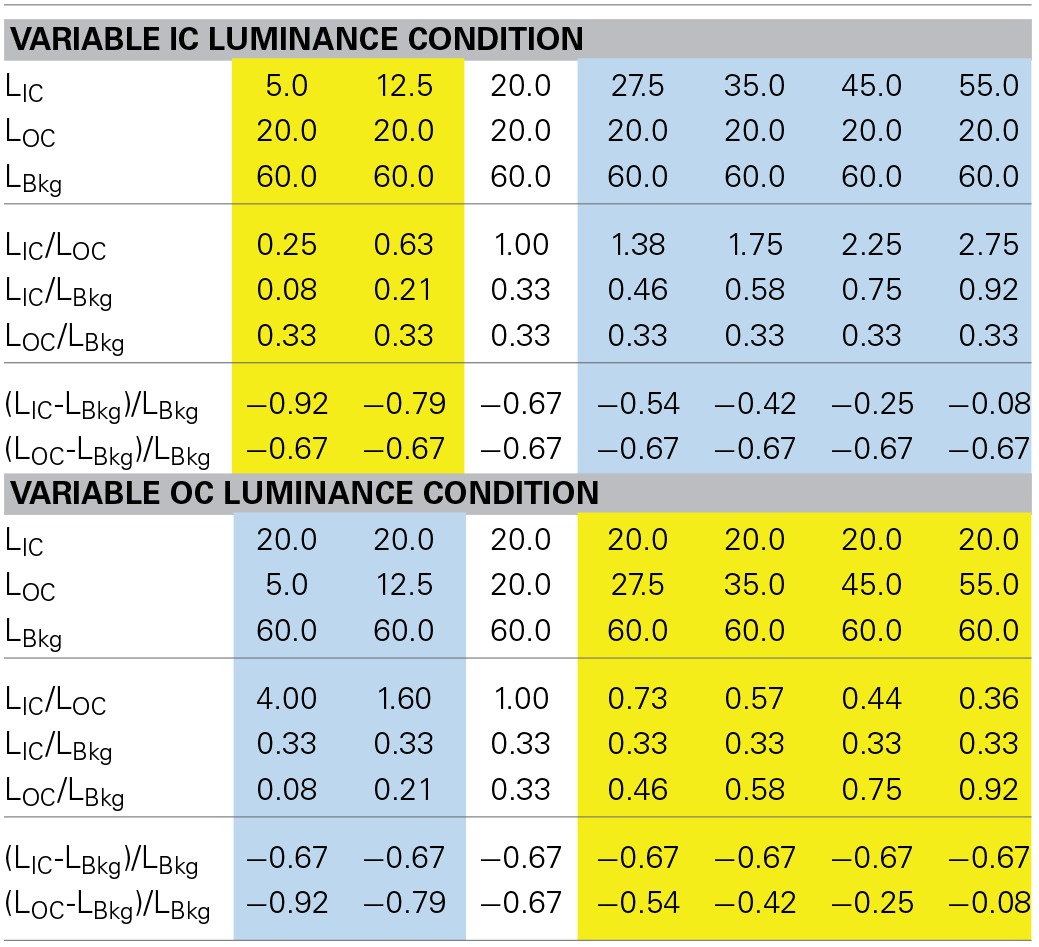
**Luminance conditions in Experiment 1**.

*Luminance values (in cd/m^2^) of different stimulus components, luminance ratios between these components, and Weber contrasts of the IC and the OC were listed. L_IC_, inner contour luminance; L_OC_, outer contour luminance; and L_Bkg_, background luminance. The Luminance conditions where the size of the Weber contrast of the IC is larger than that of the OC, i.e., |(L_IC_-L_Bkg_)/L_Bkg_| >| (L_OC_-L_Bkg_)/L_Bkg_|, are shaded yellow, whereas the ones where |(L_IC_-L_Bkg_)/L_Bkg_| <|(L_OC_-L_Bkg_)/L_Bkg_| are shaded blue*.

#### Procedure

Observers were asked to adjust the chromaticity of the test area until it appeared achromatic (a cancelation technique). The observer's adjustment was carried out by varying the stimulus along the u′ and v′ axes in the CIE u′v′ chromaticity diagram in 0.00025 step. On each trial, the chromaticity of the test area was preset to a value randomly varied from the background white (in the range of ±10 adjustment steps in u′ and v′, respectively). The step size could be made five times larger, and returned to the standard size, by pressing a key, so that observers could first roughly cancel the induced color and then adjust the color finely. The luminance of the test area was kept the same as the background luminance, because a brightness change due to color spreading was small on average in this stimulus configuration as confirmed in a preliminary experiment (see also Devinck et al., 2005).

At the beginning of each daily session, the observers dark-adapted for at least 5 min and then preadapted to the white background for 2 min. Within each session, all stimulus conditions were tested three times in a pseudo random order. Each session was repeated three times on different days and each stimulus condition was tested nine times in total for each observer. Before the experimental sessions, observers could have as many practice trials as they want to familiarize themselves with the cancelation task. However, the number of practice trials was small, as all observers had some experiences in color adjusting experiments and with the color spreading in the watercolor display.

### Results and discussion

**Figure 2:**
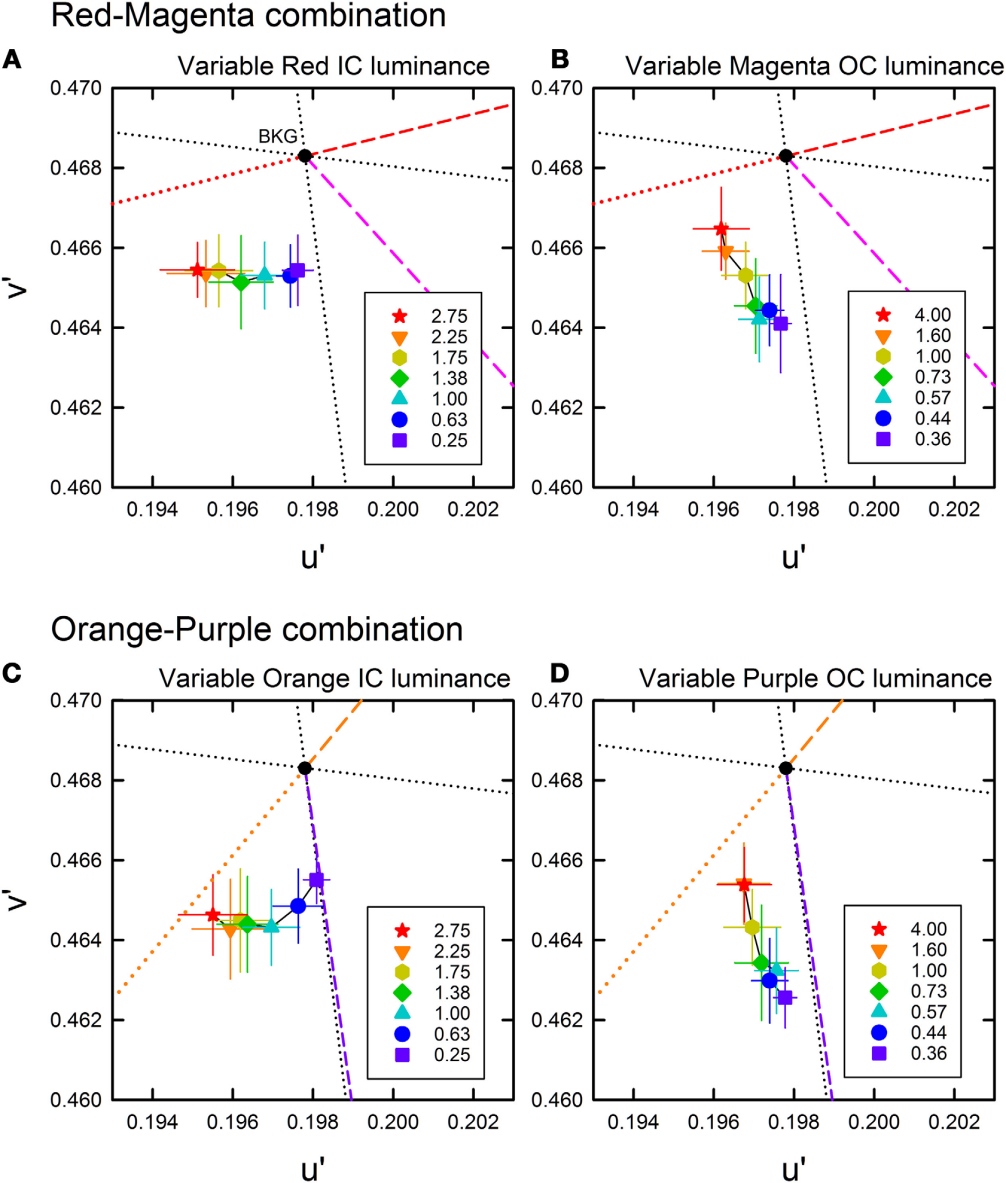
**Color cancelation data of Experiment 1 plotted in the CIE *u*′ *v*′ chromaticity diagram. (A,B)** The results for a red inner contour (IC) and magenta outer contour (OC) combination, and **(C,D)** the results for an orange IC and purple OC combination. For each color combination, the left panel **(A,C)** shows the results obtained when the IC luminance was varied and the OC luminance was fixed to 20 cd/m^2^, while the right panel **(B,D)** shows the results when the IC luminance was fixed to 20 cd/m^2^ and the OC luminance was varied (see also Table 1). Different symbols designate the mean chromaticity coordinates required to cancel chromatic induction for different IC/OC luminance ratios as shown in the legend. Error bars show ±1 s.e.m. across observers. In each panel, the direction of the IC and the OC colors are shown by colored dashed lines, which connect the chromaticity coordinates of each color and the background white (*u*′ = 0.1978, *v*′ = 0.4683; designated by the black dot). The red and orange dotted lines illustrate the complementary color direction of the IC for the red-magenta and orange-purple color combinations, respectively. If the induced color was the same as the IC color, the data symbol would have been on this line. The black dotted lines designate the (L-M) (horizontal) and the S (vertical) axes in the DKL color space (Derrington et al., 1984). Note that in the *u*′ *v*′ diagram, the upward and downward directions correspond to −S and +S directions, respectively.

The cancelation settings averaged across different observers were plotted in the CIE u′v′ chromaticity diagram (Figure 2). If the induced color had the same hue as the IC color, the chromaticity necessary to cancel it would be in the opposite direction in the chromaticity diagram (designated by red and orange dotted lines in Figure 2). A deviation to the counter clockwise direction indicates that the induced color was yellower, and a deviation to the clockwise direction indicates that the induced color was redder. The results showed that when the luminance ratio between the IC and the OC (IC/OC luminance ratio) was the largest (red star symbols in Figure 2), which is a condition suitable for assimilative color spreading (Pinna et al., 2001; Devinck et al., 2005), the cancelation setting was closest to the opposite direction to the IC color in all stimulus conditions. This result indicates the conventional assimilative watercolor effect. For the orange-purple color combination, the cancelation settings were slightly deviated from the opposite direction of the IC color to the counter clockwise direction and thus the induced color was slightly yellower than the IC color. This finding is consistent with previous findings by Devinck and colleagues. When we closely examined the color directions of cancelation settings in Devinck et al. (2005, 2006a,b), we found that they are almost always slightly deviated to the counter clockwise direction (10–20°), never to the clockwise direction. Thus, the induced color is generally a little yellower than the orange IC color. For the red-magenta color combination, the deviation to the counter clockwise direction was larger than that for the orange-purple combination, which will be discussed later.

The results also showed that the induced color systematically varied depending on the IC/OC luminance ratio. Moreover, the effects of the IC/OC luminance ratio on the induced color were very similar for both the red-magenta and the orange-purple color combinations. In both the variable IC and the variable OC luminance conditions, as the IC/OC luminance ratio became smaller from the largest values, the cancelation setting was shifted away from the opposite direction of the IC color and became closer to +S axis (or to the complementary color of the OC for the orange-purple combination) in all conditions. As the cancelation settings closer to +S axis indicate that the induced color was yellow (blue color was required to cancel it), the color spreading found with smaller IC/OC luminance ratios corresponds to non-assimilative yellow color spreading. These results are consistent with the previous findings (Devinck et al., 2005).

**Figure 3:**
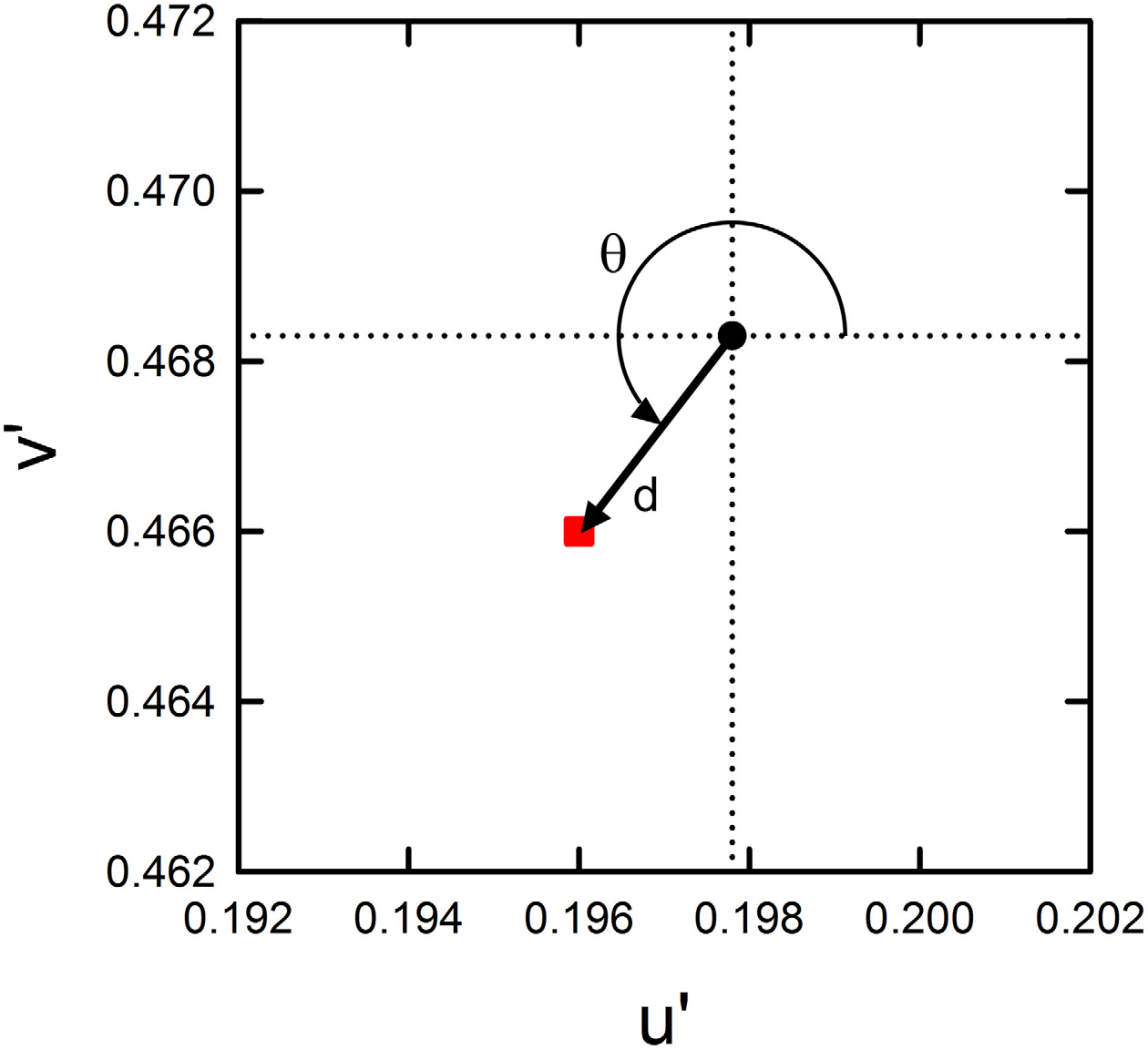
**Quantification of color cancelation data**. Using the color vector illustrated by the arrow originating at the background white (black dot) and extending to the mean setting (red square), the color direction and shift size were calculated for the mean cancelation setting. The color direction (θ) was defined as the angle from the +u′ axis to the direction of the mean setting. It should be noted that the direction is opposite to that of the perceived color. The shift size (d) was defined as the vector length (i.e., Euclidean distance) from the background white to the mean setting. When plotted in the figures (in Figures 4, **6**, **8**), it is expressed in the percentage to the Euclidean distance of the inducing contour colors (0.05).

**Figure 4:**
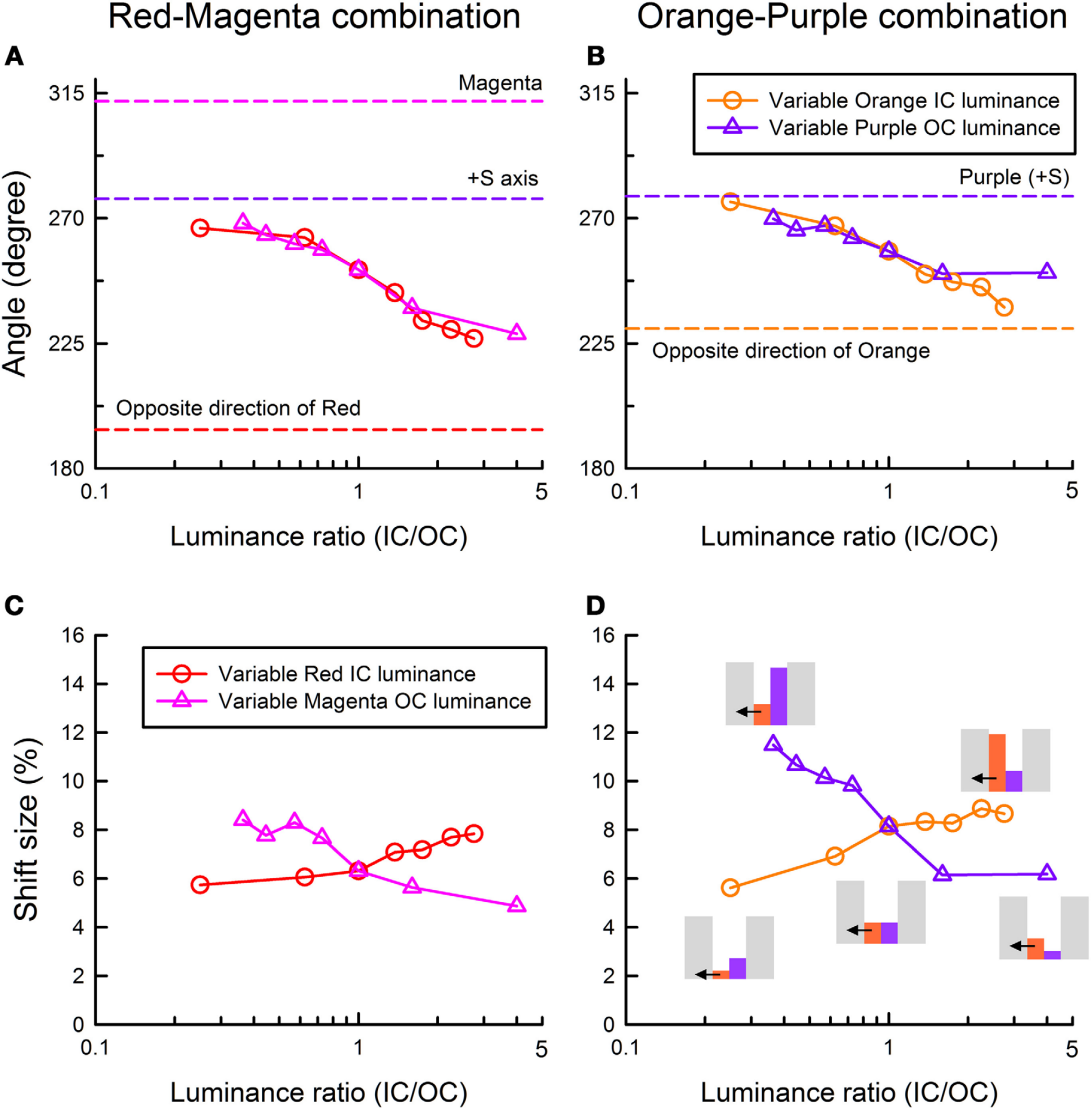
**Color direction and shift size of the cancelation settings as a function of the IC/OC luminance ratio. (A,C)** The results for the red IC and magenta OC combination, and **(B,D)** the results for the orange IC and purple OC combination. Upper panels show the results of color direction analysis, whereas lower panels show the results of shift size analysis. Circle symbols show the results in the variable IC luminance condition, while triangle symbols show those in the variable OC luminance condition. In the figures of color direction analysis **(A,B)**, important directions are also shown by dashed lines (the opposite direction of the IC color, the OC color direction and +S axis). In **(D)**, relative luminances among different stimulus regions are schematically shown as inset figures. Gray, orange, purple bars illustrate the luminances of the background, orange IC, and purple OC, respectively. Small horizontal arrows indicate the direction of color spreading that was measured in the experiment.

To further quantify the changes in the induced color, we calculated the color direction and shift size of the mean settings (Figure 3). The color direction was defined as the angle from the +u′ axis to the direction of the mean cancelation setting. If the induced color had the same hue as the IC color, the color direction would be 194.0° for the red-magenta color combination and 230.4° for the orange-purple combination. The magnitude of color spreading was quantified with the shift size defined as the distance to the mean cancelation setting from the background white point, expressed in the percentage to the Euclidean distance of the inducing contour colors (0.05). The results of the color direction analysis (Figures 4A,B) show that changes with the IC/OC luminance ratio were very similar under the variable IC and the variable OC luminance conditions (circles and triangles in each graph, respectively). When plotted in the same graph, the results under the two conditions were almost superimposed on each other. Thus, the IC/OC luminance ratio seems to determine the hue of the induced color. Moreover, the color direction of the cancelation settings was in between the opposite direction of the IC color and the OC color, indicating that the induced color can be described as an additive mixture of the IC color and the complementary color of the OC. These results also showed that the range of the induced color was more limited for the red-magenta combination (Figure 4A) than for the orange-purple combination (Figure 4B). For the orange-purple combination, the induced color changed from the one similar to the IC color to the one complementary to the OC color depending on the IC/OC luminance ratio (Figure 4B). However, for the red-magenta combination (Figure 4A), when the IC/OC luminance ratio was the largest, the induced color was close to red but deviated to the yellow direction (about 30°). When the IC/OC ratio was small, the color direction was close to +S axis and thus the induced color was yellow, rather than the complementary color of magenta (yellow-green; corresponding to a dashed line labeled “Magenta”). The deviation from the complementary color was about 45°. These possible differences in the range of the induced color direction may reflect the effects of color combination on assimilative and non-assimilative spreading. Differential effects of color combination on assimilative color spreading have been reported in previous watercolor studies (Devinck et al., 2006b; Coia et al., 2014). As the complementary color of the OC for the orange-purple combination was yellow, the non-assimilative color spreading in the present stimulus configuration can be summarized as yellow spreading.

The results of the shift size (Figures 4C,D) showed that the magnitude of color spreading varied differently with the IC/OC luminance ratio in the variable IC and the variable OC luminance conditions. In the variable IC condition, larger IC/OC luminance ratios produced stronger color spreading (circles in Figures 4C,D). In contrast, in the variable OC luminance conditions, smaller IC/OC luminance ratios produced stronger color spreading (triangles in Figures 4C,D). Previous studies showed that the spreading was very weak when the OC luminance was in between the IC and the background luminance (Devinck et al., 2005; Cao et al., 2011). The present study showed that this is true only for the results in the variable IC luminance conditions (two left circles in Figures 4C,D). The reverse is the case for the results in the variable OC luminance conditions (four left triangles in Figures 4C,D), although the spreading was non-assimilative.

The present results confirmed that luminance conditions play a critical role in determining the induced color in the watercolor effect as suggested by previous studies (Devinck et al., 2005; Kitaoka, 2007). Moreover, the present study revealed that the color direction and magnitude of the color spreading differently depended on the luminance condition (Figure 4). The results suggested that the luminance condition suitable for assimilative color spreading is different from that for non-assimilative color spreading and that the induced color is determined by relative contributions of the assimilative and non-assimilative color spreading. The assimilative color spreading became stronger with larger IC/OC luminance ratios in the variable IC luminance condition, whereas the non-assimilative color spreading became stronger with smaller IC/OC ratios in the variable OC luminance condition.

To further characterize the luminance conditions for different types of color spreading, the effects of the background luminance was investigated in Experiment 2. As the background luminance was fixed in Experiment 1, the IC/OC luminance ratio covaried with the luminance ratio between the contour and the background, and with the Weber contrast of the IC and the OC (Table 1). Experiment 2 aimed to differentiate the effects of these luminance measures.

## Experiment 2

In Experiment 2, we investigated color spreading in the watercolor configuration by manipulating the background luminance while keeping the IC/OC luminance ratio constant.

### Methods

#### Observers

The same three observers who had participated in Experiment 1 also participated in Experiment 2.

#### Stimuli and procedure

The same combinations of the IC and OC colors as in Experiment 1 were also used in Experiment 2. The chromaticity coordinate of orange was slightly changed to *u*′ = 0.2324, *v*′ = 0.5045 to deal with a technical issue. The background luminance was manipulated from 5 to 60 cd/m^2^. At each background luminance, two contour luminance conditions were tested for both the red-magenta and the orange-purple combination. In the red (orange) darker IC condition, the IC luminance was 20 cd/m^2^ and the magenta (purple) OC luminance was 45 cd/m^2^. In the red (orange) lighter IC condition, the IC luminance was 45 cd/m^2^ and the magenta (purple) OC luminance was 20 cd/m^2^ (Table 2).

**Table 2:**
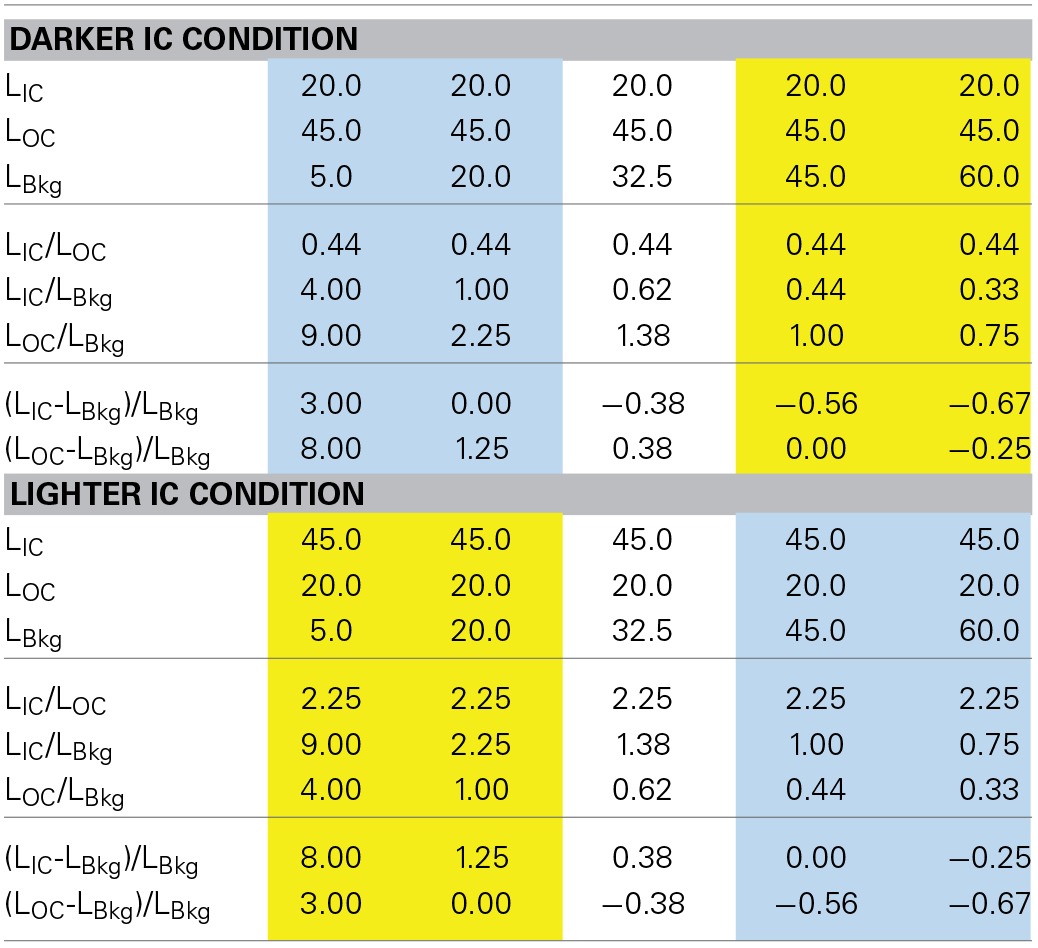
**Luminance conditions in Experiment 2**.

*Luminance values (in cd/m^2^) of different stimulus components, luminance ratios between these components, and Weber contrasts of the IC and the OC were listed. L_IC_, inner contour luminance; L_OC_, outer contour luminance; and L_Bkg_, background luminance. The Luminance conditions where |(L_IC_-L_Bkg_)/L_Bkg_| >| (L_OC_-L_Bkg_)/L_Bkg_| are shaded yellow, whereas the ones where |(L_IC_-L_Bkg_)/L_Bkg_| <|(L_OC_-L_Bkg_)/L_Bkg_| are shaded blue*.

The observers dark-adapted for at least 5 min at the beginning of each daily session and then all stimulus conditions were tested five times in a pseudo random order at all five background luminances. The measurement at each background luminance was blocked and the background luminance was chosen in ascending order. Before the measurement at a different background luminance, the observers adapted to the white background for 2 min. Within each session, all stimulus conditions were tested five times in a pseudo random order. Each session was repeated twice on different days and each stimulus condition was tested 10 times in total for each observer. All other aspects of the method were the same as those in Experiment 1.

### Results and discussion

**Figure 5:**
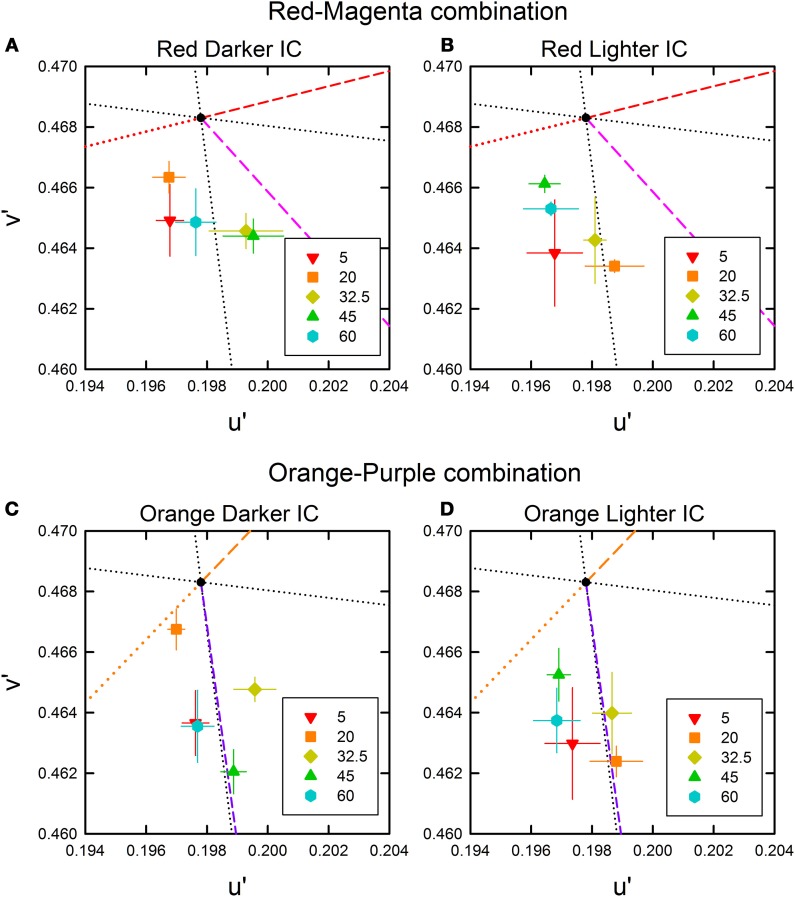
**Color cancelation data of Experiment 2 plotted in the CIE u′v′ chromaticity diagram. (A,B)** The results for the red IC and magenta OC combination, and **(C,D)** the results for the orange IC and purple OC combination. For each color combination, the left panel **(A,C)** shows the results obtained when the IC luminance was darker (20 cd/m^2^) and the OC luminance was lighter (45 cd/m^2^), while the right panel **(B,D)** shows the results when the IC luminance was lighter (45 cd/m^2^) and the OC luminance was darker (20 cd/m^2^) (see also Table 2). Different symbols designate the mean chromaticity coordinates required to cancel chromatic induction for different background luminances (in cd/m^2^) as shown in the legend. Other aspects are the same as in Figure 2.

**Figure 6:**
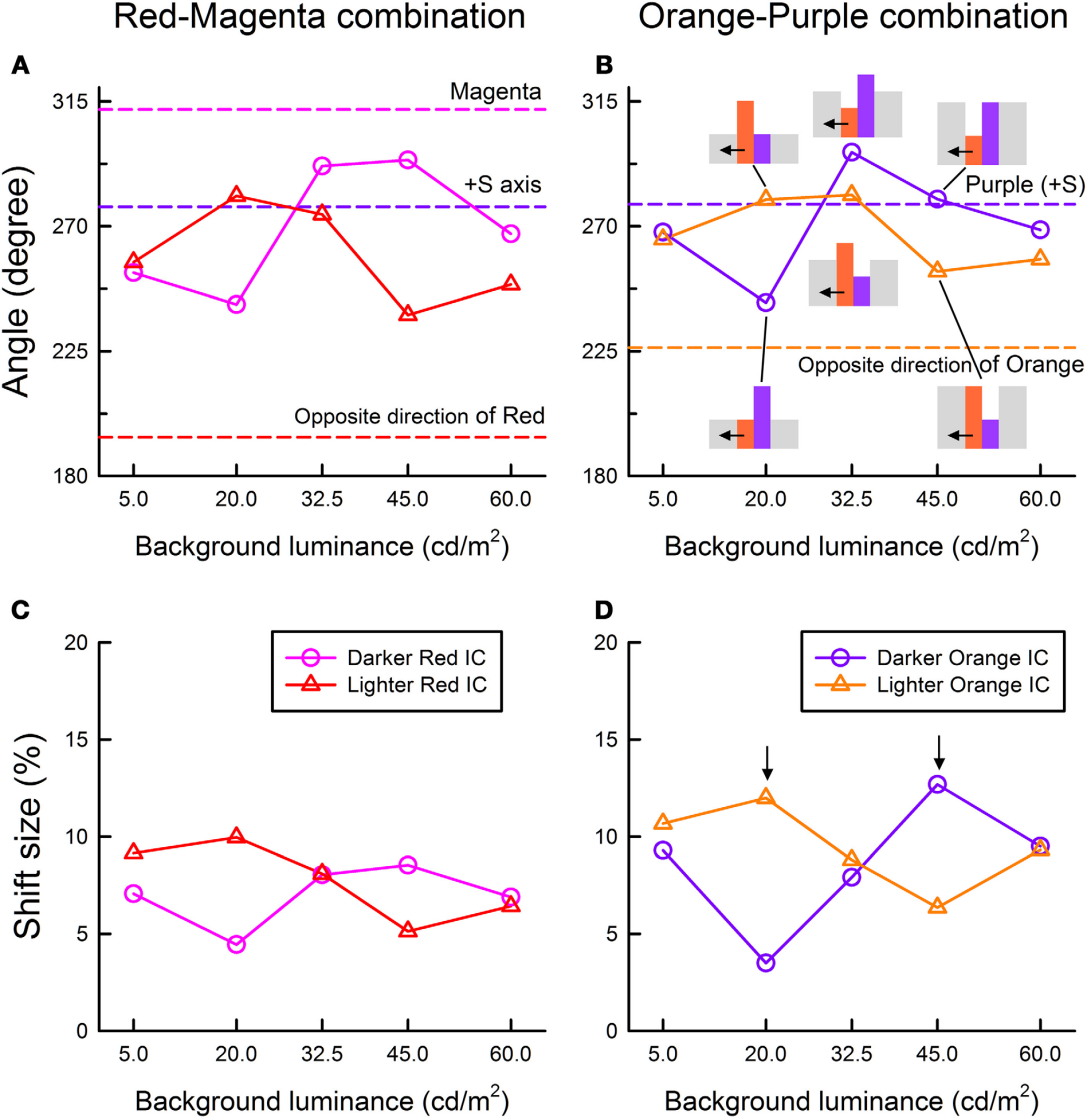
**Color direction and shift size of the cancelation settings as a function of the background luminance. (A,C)** The results for the red IC and magenta OC combination, and **(B,D)** the results for the orange IC and purple OC combination. Upper panels show the results of color direction analysis, whereas lower panels show the results of shift size analysis. Circle symbols show the results in the darker IC condition (the IC and the OC luminance were 20 and 45 cd/m^2^, respectively), while triangle symbols show those in the lighter IC condition (the IC and the OC luminance were 45 and 20 cd/m^2^, respectively). In **(B)**, relative luminances among different stimulus regions are schematically shown as inset figures. Other aspects are the same as in Figure 4.

The cancelation settings averaged across different observers were plotted in the CIE u′v′ chromaticity diagram (Figure 5). Results showed that color spreading could be observed at all background luminances (Pinna et al., 2001; Pinna and Reeves, 2006). Moreover, the results revealed an important interaction between the contour luminances and the background luminance, which can be more clearly seen in the results of color direction analysis in Figures 6A,B. Even when the IC and the OC luminances and thus the IC/OC luminance ratio were all fixed, the induced color systematically varied with the background luminance. Thus, the relative luminance of the contours to the background plays a critical role in determining the induced color in the watercolor configuration. The luminance relations among the IC, OC and background were schematically illustrated by inset figures in Figure 6B (see also Table 2). For both the red-magenta and the orange-purple color combinations, the induced color became similar to the IC color when the background luminance was similar to the IC luminance. On the other hand, when the background luminance was similar to the OC luminance or at the midpoint between the IC and the OC luminances, the induced color became yellow (or close to the complementary color of the OC for the orange-purple combination). It should be noted that these tendencies hold true regardless of the contour luminance conditions (circle and triangle symbols in Figures 6A,B). Thus, the sign (or polarity) of luminance differences is not important.

The results of the shift size (Figures 6C,D) indicate that maximal color spreading was observed when the background luminance was the same as the OC luminance and the color spreading was non-assimilative (designated by downward arrows in Figure 6D). Thus, non-assimilative spreading can be stronger than conventional assimilative spreading. Supplementary Figure 1 demonstrates the effects of the background luminance on the non-assimilative color spreading for a red-magenta combination.

The results of Experiments 1 and 2 showed that the luminance conditions suitable for assimilative color spreading are different from those for non-assimilative color spreading. However, these luminance conditions cannot be simply described in terms of the IC/OC luminance ratio, nor in terms of the ratio of the contour to the background luminances. The OC to background luminance ratio was always larger than the IC to background luminance ratio in the darker IC condition, while the reverse relation always held in the lighter IC condition in Experiment 2. We found that the results can be summarized reasonably well in terms of the Weber contrast of the IC and the background luminance (IC contrast) and that of the OC and the background luminance (OC contrast) (Tables 1, 2). That is, when the size (or absolute value) of the IC contrast was smaller than the OC contrast (blue shaded conditions in Tables 1, 2), assimilative color spreading was observed, although some exceptions were found such as the lowest background luminance in the darker IC luminance condition and the highest luminance condition in the lighter IC luminance condition (Table 2). This luminance condition for assimilative color spreading is consistent with that for color diffusion from the IC proposed in the model of Grossberg and Mingolla (1985). On the other hand, when the luminance condition was opposite, that is, when the size of the IC contrast was *larger* than the OC contrast (yellow shaded conditions in Tables 1, 2), non-assimilative color spreading was observed and the induced color was close to yellow. It should be noted that according to the model of Grossberg and Mingolla (1985) this luminance condition would produce *assimilative* color spreading in the region delineated by the *OC* of lower contrast, not by the IC of higher contrast. However, what was actually observed was non-assimilative yellow color spreading in the region delineated by the IC. These results suggest that the non-assimilative color spreading is at least partially mediated by a different mechanism from that underlying the assimilative color spreading.

Experiment 3 further investigated the effects of the luminance conditions on color spreading. Although the watercolor effect has been characterized as unilateral color spreading, if the luminance conditions suitable for the assimilative and the non-assimilative color spreading are opposite, bilateral color spreading could be observed when the stimulus is appropriately set up. This prediction was tested in Experiment 3.

## Experiment 3

Experiment 3 investigates the possibility of bilateral color spreading in the watercolor configuration.

### Methods

#### Observers

Four observers participated in Experiment 3. Two of them (including the second author) also participated in the previous experiments. Two new observers had normal or corrected-to-normal visual acuity and normal color vision, and were naïve regarding the purpose of the experiment.

#### Stimuli and procedure

To reduce spatial asymmetry for different directions of color spreading, we used a four-column configuration (Figure 1E) in Experiment 3. The stimulus subtended 3.8 × 4.8° and the contours were sinusoidally shaped at 1.9 cycles per degree (0.4° peak-to-trough amplitude and 4.5 min thick). The color combinations were the same as in Experiments 1 and 2. The background luminance was 60 cd/m^2^. Three different contour luminance conditions were tested. The luminance of one contour was 20 cd/m^2^ and that of the other was 45 cd/m^2^ in one condition, and vice versa in another condition. In the third condition, the luminance of both contours was 32.5 cd/m^2^. These conditions were designated for the red-magenta combination as “R20/M45” (the luminance of red color was 20 cd/m^2^ and that of magenta was 45 cd/m^2^), “R45/M20,” “R33/M33,” respectively. Similar notations were also used for the orange-purple combination.

On each trial, the observers were asked to cancel the color spreading in the first and the third columns (or in the second or forth columns) in the stimulus. Which columns to be canceled was designated by a small black dot presented a little above one of the columns. Other than the observers' adjustment changed the colors in the two columns simultaneously, the cancelation procedure was the same as in Experiments 1 and 2.

The observers dark-adapted for at least 5 min at the beginning of each daily session and then preadapted to the white background for 2 min. Within each session, all stimulus conditions were tested three times in a pseudo random order. Each session was repeated twice on different days and each stimulus condition was tested six times in total for each observer. All other aspects of the method were the same as those in Experiment 1.

### Results and discussion

**Figure 7:**
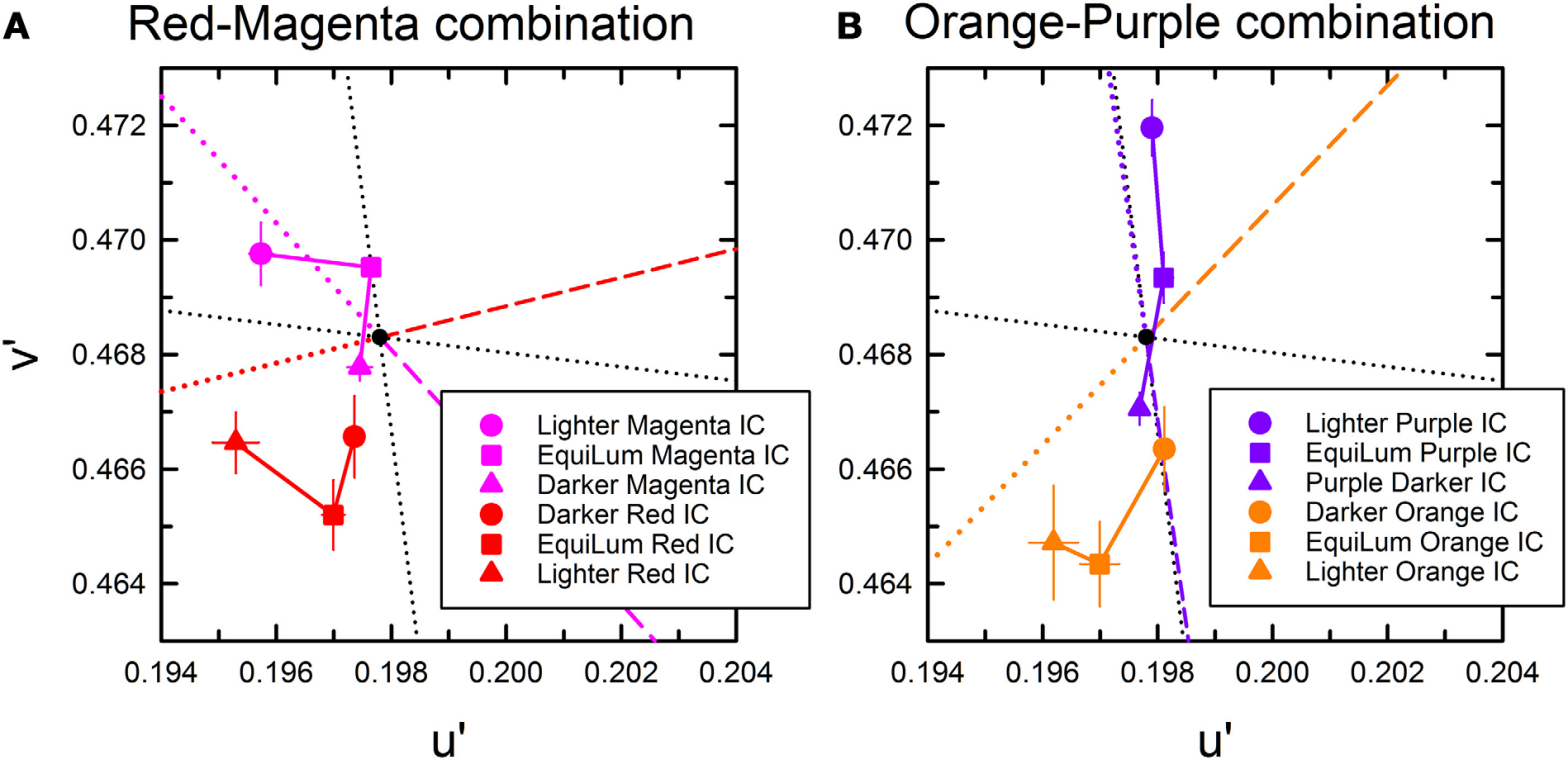
**Results of Experiment 3 plotted in the CIE u′v′ chromaticity diagram. (A)** The results for the red and magenta combination and **(B)** those for the orange and purple combination. Different symbols are associated with different contour luminance conditions. In **(A)**, circles: R20/M45 (the luminance of red color was 20 cd/m^2^ and that of magenta was 45 cd/m^2^), squares: R33/M33 (red: 32.5 cd/m^2^ and magenta: 32.5 cd/m^2^), and triangles: R45/M20 (red: 45 cd/m^2^ and magenta: 20 cd/m^2^) conditions. In **(B)**, circles: O20/P45, squares: O33/P33, and triangles: O45/P20 conditions. The symbol color corresponds to the IC color enclosing the region in which color spreading was measured as shown in the legend (see also inset illustrations in Figures 8C,D). Other aspects are the same as in Figure 2.

**Figure 8:**
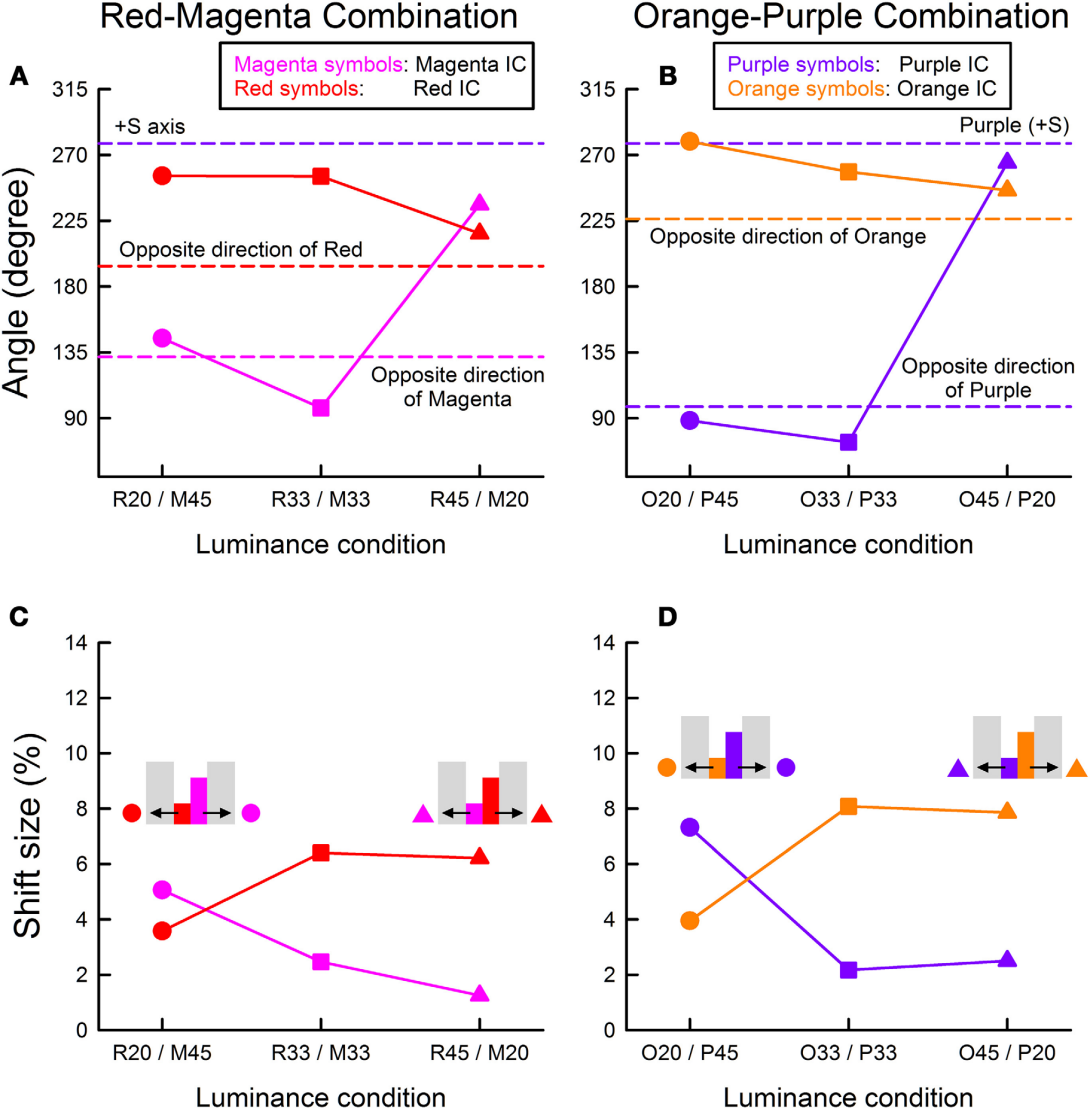
**Color direction and shift size of the cancelation settings as a function of the contour luminance condition. (A,C)** The results for the red and magenta combination, and **(B,D)** those for the orange and purple combination. Upper panels show the results of color direction analysis, whereas lower panels show the results of shift size analysis. The same symbol color and form are used as in Figure 7, and thus the color and form correspond to the IC color and relative contour luminance, respectively. Inset illustrations in Figures 8C,D show which symbols correspond to which color spreading. Other aspects are the same as in Figure 4.

The cancelation settings averaged across different observers were plotted in the CIE u′v′ chromaticity diagram (Figure 7). The results of color direction and shift size analyses were shown in Figure 8. In the R20/M45 condition, two of the four columns were delineated by the lighter magenta IC and darker red OC (magenta circle in Figures 7A, 8A; also see an inset illustration in Figure 8C), while the other two columns were delineated by the darker red IC and lighter magenta OC (red circle). The size of the IC contrast was smaller than that of the OC contrast for the former two columns and thus, based on the results in the previous experiments, assimilative color spreading was expected in these columns. In contrast, for the latter two columns, the size of the IC contrast was larger than that of the OC contrast and thus non-assimilative color spreading was expected. The results were consistent with these predictions. The induced color in the columns delineated by the lighter magenta IC and darker red OC (magenta circle) was closer to magenta color (i.e., the color direction of the cancelation setting was close to the complementary color of magenta; Figure 8A). The induced color in the columns delineated by the darker red IC and lighter magenta OC (red circle) was yellow (i.e., the color direction of the cancelation setting was close to +S axis). Results also showed that the assimilative color spreading was stronger than the non-assimilative yellow color spreading (Figures 7A, 8C). Overall, assimilative color spreading was induced on one side, and non-assimilative spreading was induced on the other side, and thus bilateral color spreading was found in the four-column configuration. Similar results were found in the O20/P45 condition for the orange-purple combination (orange and purple circles in Figures 7B, 8B,D).

A different pattern of results was found in the R45/M20 condition. In this condition, two of the four columns were delineated by the lighter red IC and darker magenta OC (red triangle in Figures 7A, 8A; also see an inset illustration in Figure 8C), while the other two columns were delineated by the darker magenta IC and lighter red OC (magenta triangle). In the former two columns (red triangle), red assimilative color spreading was found, although the induced color was again yellower than the IC color. This result is consistent with those in Experiment 1. Thus, taken together with the results in the R20/M45 condition, for the assimilative color spreading (magenta circle and red triangle), the luminance condition was critical, and interchanging the IC and the OC colors did not affect the strength of the color spreading; it simply changed the induced color which was similar to the IC color. In contrast, in the columns delineated by the darker IC and lighter OC, the strength of the color spreading changed by interchanging the IC and the OC colors (red circle and magenta triangle). As just described above, when the IC was dark red and the OC was light magenta (red circle), non-assimilative color spreading of moderate magnitude was observed. However, interchanging colors much reduced the color spreading (magenta triangle). Thus, for the non-assimilative color spreading, the color condition as well as the luminance condition were critical. Because of the small size of the color induction, the color direction of the cancelation setting was not clear. But it appears similar to the one induced on the other side of contours; the magenta triangle was located close to the red triangle in Figure 8A. These results are not consistent with the hypothesis that the induced color in the watercolor configuration is described as an additive mixture between the IC color and the complementary color of the OC.

The results in the R33/M33 condition (red and magenta squares) were like an intermixture of the results in the R20/M45 and in the R45/M20 conditions. The color direction in the R33/M33 condition was close to that in the R20/M45 condition, but the magnitude of the spreading was similar to that in the R45/M20 condition. The results were similar for the orange-purple combination (orange and purple squares).

Some readers may raise a possibility of simultaneous color contrast to account for the results of Experiment 3. That is, a color induced on one side of the double contours may have, in turn, induced its complementary color on the other side of the contours. To rule out this possibility, we conducted an additional cancelation experiment where the induced colors in all four columns of the stimulus were canceled. The results were almost the same as those obtained in Experiment 3. Thus, even after the induced color in the first and third columns were canceled, the observers could still observe color spreading in the second and forth columns (and vice versa). The results of this additional experiment also supported bilateral color spreading.

## General discussion

This study explored the effects of the luminance conditions on color spreading in the watercolor configuration. We confirmed that the color spreading can be accounted for by two components; one is assimilative, and the other is non-assimilative. The former component diffused the color of the IC into the region enclosed by the IC and contributed more strongly to color spreading when the Weber contrast of the IC to the background luminance (IC contrast) was smaller in size than that of the OC (OC contrast). In contrast, the latter non-assimilative component has several interesting properties. It was associated with the features of the OC but induced a color into the region delineated by the IC (Pinna, 2006). The induced color was yellow (or similar to the complementary color of the OC for the orange-purple combination). Moreover, it contributed more strongly to color spreading when the OC contrast was smaller than the IC contrast. Thus, the luminance condition suitable for the non-assimilative color spreading was opposite to that for the assimilative color spreading. This caused systematic changes in apparent hue of the induced color with the IC/OC luminance ratio, which was observed in Experiment 1 as well as in Devinck et al. (2005). Moreover, the opposite suitable luminance conditions could lead to bilateral color spreading (Experiment 3); assimilative color spreading on one side of the double contours and non-assimilative color spreading on the other side. Finally, the non-assimilative component can be specific to color conditions as shown in Experiment 3; a combination of a dark red IC and light magenta OC (or of a dark orange IC and light purple OC) induced non-assimilative spreading, whereas when the contour colors were interchanged, i.e., for a combination of a dark magenta IC and light red OC (or of a dark purple IC and light orange OC), color spreading was much reduced. This finding was specific to non-assimilative color spreading; in assimilative color spreading, the same manipulation caused color spreading of different hue but of similar magnitude. Overall, these results suggest that the assimilative and the non-assimilative components of color spreading are at least partially mediated by different visual mechanisms^1^.

The model proposed by Grossberg and Mingolla (1985) assumes a weakening of low-contrast boundaries through spatial competition by high-contrast boundaries for letting a color flow out through boundaries. Recently, Cao et al. (2011) investigated luminance conditions for the achromatic watercolor effect and confirmed achromatic diffusion from low-contrast boundaries and insensitivity of spatial competition to the contrast polarity. Spreading was small but measurable when the size of the OC contrast was large regardless of the sign of the contrast. When the OC luminance was in between the IC and the background luminance and thus the OC contrast was smaller than the IC contrast, the spreading mostly disappeared. The present findings regarding the effects of luminance conditions on assimilative color spreading can be accounted for by the color diffusion from low-contrast boundaries assumed in the model by Grossberg and Mingolla (1985), and the present results also confirmed the insensitivity of color spreading to contrast polarity (Figure 6). Overall, the luminance conditions suitable for assimilative spreading were consistent with those reported by previous studies (Pinna et al., 2001; Devinck et al., 2005; Cao et al., 2011).

For non-assimilative color spreading, we need to consider the elaboration of the model by Bressan (1995) which involves complementary color induction from the OC to the IC if we apply it to the watercolor display. Although the induced color of non-assimilative color spreading in the watercolor configuration is more or less similar to the prediction, the contour luminance contrast is not right for the proposed color induction. In the model, the complementary color of the OC would be induced from the OC to the IC as a manifestation of simultaneous color contrast and supposed to flow out through weakened low-contrast boundaries between the IC and the background. However, the present results were opposite to the prediction; non-assimilative color spreading was larger when the size of IC contrast was *larger* than that of the OC contrast (Figures 4, 6). The luminance conditions suitable for the non-assimilative spreading were also inconsistent with the findings by Cao et al. (2011). When the OC luminance was in between the IC and the background luminance, the non-assimilative spreading can be stronger (e.g., Figure 4D). When empirically testing Bressan's (1995) proposal, da Pos and Bressan (2003) suggested that the luminance difference between lattice and segments in the neon color configuration is not critical for producing color spreading. For example, in their experiments using different combinations of colored lattices and achromatic segments, complementary color spreading was observed either when the segments had higher luminance or when the segments had lower luminance, depending on the color of the lattice. da Pos and Bressan (2003) argued that the lattices need not have a larger luminance difference than segments and ascribed the stronger inhibition of the lattices on segments to spatial factors; i.e., segments are shorter than lattices and thus weakened by lattices on both ends. Similar spatial factors may also work in the watercolor configuration, because we noticed that bilateral color spreading is more easily observed in the four-column configuration used in Experiment 3 than in the two-square configuration used in Experiments 1 and 2. However, the spatial factors cannot account for the clear dependency of the non-assimilative color spreading on luminance conditions.

Because of asymmetric spatial competition between boundaries, Grossberg and Mingolla's (1985) model generally predicts unilateral color spreading. However, a weak bilateral color spreading has been observed by Pinna et al. (2001) when an orange IC and purple OC were equiluminant. Pinna et al. (2001) argued that at equiluminance purple/white and orange/white edges mutually inhibit each other and thus produce a weak bilateral effect. The effect was assimilative on either side of the double contours. Takashima (2008) also reported bilateral spreading in the achromatic watercolor display when the background luminance was in between the IC and the OC luminance. The white-black double contours on a gray background produced light spreading on the side of the white contour and dark spreading on the side of black contour (see Figure 6 in Takashima, 2008 for a demonstration of the effect). This another bilateral effect may also be accounted for by mutual inhibition between boundaries, because the spreading was also assimilative on either side of the double contours. The present study found bilateral color spreading when the IC and the OC luminance were not similar to each other (circle symbols in Figure 8). An important difference from the previous findings is that, in the present study, the color spreading was assimilative on one side but non-assimilative on the other side. For example, when the four-column stimulus was composed of a lighter magenta and darker red contours, the color spreading on the magenta side was assimilative (magenta) while the spreading on the red side was non-assimilative (yellow). This new bilateral color spreading cannot be accounted for by symmetric spatial competition between boundaries. Thus, visual mechanisms other than color diffusion from weakened boundaries are necessary to account for the non-assimilative color spreading. The present study also found that when the IC and the OC were equiluminant the bilateral color spreading was measurable but the effect on one side was weak (square symbols in Figure 8). For symmetric bilateral color effects at equiluminance, the luminance contrast of the double contours may need to be smaller.

This study focused on luminance conditions for color spreading in the watercolor configuration, but some results are relevant to color processing underlying color spreading. Firstly, Experiment 1 showed that the apparent hue of assimilative color spreading was yellower than the IC color and the size of the deviation to the yellow direction can be different for different color combinations. Similar yellower spreading was also observed in previous studies (Devinck et al., 2005, 2006a,b). One possible cause of this yellower spreading is the contribution of non-assimilative yellow spreading. The non-assimilative contribution may always be found in the watercolor effect. Another possible cause is the effect of chromatic adaptation. Cao et al. (2011) discussed that the weak spreading on a large surface as in the watercolor effect can generate rapid adaptation effects. Adaptation can work to desaturate the induced color. Investigating this issue may be difficult in some cases, because when the color spreading is not very strong, the apparent hue of the induced color can be ambiguous. As a promising solution, one of the reviewers suggested restricting the nulling chromatic axis in the cancelation task. If the induced color cannot be canceled when the nulling axis is restricted to the complementary color of the IC, it indicates that the deviation to the yellow direction reflects actual hue shift of the induced color.

Secondly, the apparent hue of the non-assimilative color spreading needs to be discussed, because the present study showed that the induced color in the non-assimilative color spreading was generally yellow. This induced hue was very similar to the complementary color of the OC for the orange IC and purple OC combination. However, for the red IC and magenta OC combination, the hue was largely shifted from the complementary color of the OC, which is yellow-green. Only in a few conditions, the induced color became closer to yellow-green (see diamond and triangle symbols in Figure 5A). These findings appear contradictory to the previous findings by Pinna (2006) that the spreading of the complementary color of the OC was observed when the IC was achromatic and the OC was chromatic. This apparent difference may be accounted for by differences in stimulus conditions. A notable difference was the saturation of the contour color; Pinna (2006) used much saturated contour colors compared to the present colors (2–4 times more saturated in terms of the Euclidean distance in the u′v′ chromaticity diagram, and the saturation was larger for green, yellow, and red than for blue). Another relevant difference can be the observation mode; the stimulus was drawn on a white paper in Pinna (2006).

Although the present results do not support a particular mechanism mediating the non-assimilative color spreading, our recent studies with similar stimuli suggest that S-cone mechanisms play a critical role in producing non-assimilative color spreading (Kuroki and Kimura, 2012). Particularly, for the non-assimilative color spreading to occur, +S contrast of the OC to the IC seems to be important. Consistently, in the color combination for which larger non-assimilative color spreading is observed, the OC is always bluer than the IC; e.g., black (IC)-blue (OC), red-magenta, green-cyan, and orange-purple color combinations. Moreover, the observation that the color induced for these combinations is generally yellow may also be related to the contribution of S-cone mechanisms. These observations are in marked contrast to the previous findings that the contribution of L-M mechanisms was larger than that of S-cone mechanisms to assimilative color spreading (Devinck et al., 2006b). Further studies are necessary to investigate the contributions of cone-opponent mechanisms to both assimilative and non-assimilative color spreading in the watercolor effect.

To sum up, the present studies revealed that assimilative and non-assimilative color spreading in the watercolor effect exhibit different properties. Notable ones are that the luminance conditions suitable for the two types of spreading are nearly opposite and that different types of spreading can be induced on different sides of the double contours in the watercolor configuration. The watercolor effect has been regarded as a good example to investigate the interaction of visual mechanisms underlying boundary and surface perception (Pinna et al., 2001; Pinna and Grossberg, 2005; Cao et al., 2011). The present findings suggest that the interaction may be more complex than previously thought.

### Conflict of interest statement

The authors declare that the research was conducted in the absence of any commercial or financial relationships that could be construed as a potential conflict of interest.

## Footnotes

^1^As a possible additional difference between assimilative and non-assimilative color spreading, we noticed that in some watercolor displays of luminance conditions suitable for non-assimilative color spreading, color induction similar to neon flank can be observed. That is, the induction is like yellow fog and confined to the vicinity of the boundary between the IC and the background. We have never observed a similar phenomenon under the stimulus conditions suitable for assimilative color spreading used in this study. However, as Pinna and Grossberg (2005) showed that color spreading in the watercolor configuration can exhibit different phenomenal appearances depending on spatial context, more systematic investigation will be necessary.
