# Supplementary material for: Assimilative and non-assimilative color spreading in the watercolor configuration
Source: Front Hum Neurosci. 2014 Sep 19;8:722. doi: 10.3389/fnhum.2014.00722 (PMC4168700; doi:10.3389/fnhum.2014.00722)
Supplement: Supplementary file 1 [file Presentation1.PDF]

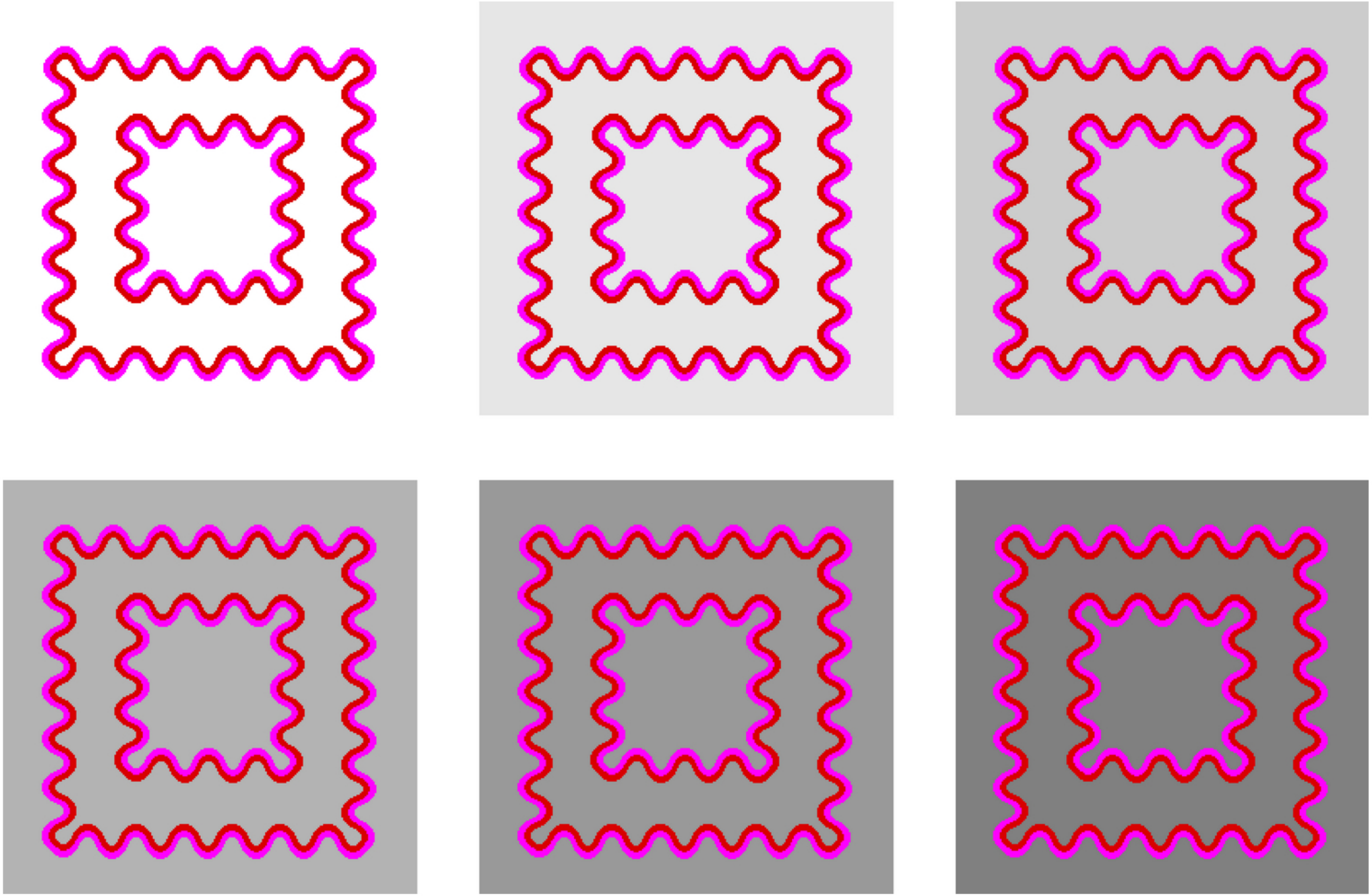

Supplementary Figure 1: Effects of background luminance on non-assimilative color spreading. Although the luminances of the red and magenta contours are fixed, the magnitude of spreading (i.e., strength of yellow color in the corridor region) would be larger when the background luminance is similar to the outer contour luminance (possibly the upper right or lower left figure, depending on the monitor setup).
